# 2201. Impact of Educational Sessions on Knowledge and Attitudes About RSV Infection and Prevention Among Residents of Long-Term Care Facilities

**DOI:** 10.1093/ofid/ofac492.1820

**Published:** 2022-12-15

**Authors:** Stefan Gravenstein, Jamison Feramisco, Jeffrey D Carter, Laura Simone, Marykate Nelson

**Affiliations:** Warren Alpert Medical School of Brown University, Providence, Rhode Island; Advanced Healthcare Solutions, Dallas, Texas; PRIME Education, LLC, Fort Lauderdale, Florida; PRIME Education, LLC, Fort Lauderdale, Florida; PRIME Education, Drexel Hill, Pennsylvania

## Abstract

**Background:**

Older adults are at risk for severe disease following respiratory syncytial virus (RSV) infection. We assessed knowledge and attitudes regarding RSV infection and prevention among residents of long-term care facilities (LTCFs).

**Methods:**

Between October and December of 2021, surveys were administered to residents of LTCFs before and after 1.25-hour educational sessions led by health care professionals (HCPs) at 5 centers.

**Results:**

Surveys were completed by 167 residents (76% female, average age 67 years). Residents reported low levels of knowledge of RSV-related topics, which increased significantly after the sessions (Figure 1). Before the sessions, 43% used family/friends and social media to obtain information about RSV and other infectious diseases, while only one-fourth consulted an HCP to learn about these topics. After the sessions, residents were more likely to confer with their HCP (*P* < .001) and less likely to utilize unreliable sources.

Although most residents indicated that they would get tested as soon as possible if they came down with symptoms suggestive of RSV or other infections, only 64% reported high confidence in preventing spread. Although most residents believed that it was important to get vaccinated against respiratory viruses and that FDA-approved vaccines are safe, only 48% would be very willing to get an RSV vaccine if available. This proportion increased to 74% after the educational sessions (Figure 2). Less than one-half were very comfortable sharing questions and concerns about vaccines with HCPs, which increased to 62% after the sessions (*P* = .016). After attending the sessions, more than 80% reported that they plan to get an RSV vaccine when one is available and be more diligent in taking actions to prevent the spread of infectious diseases.

**Figure:**
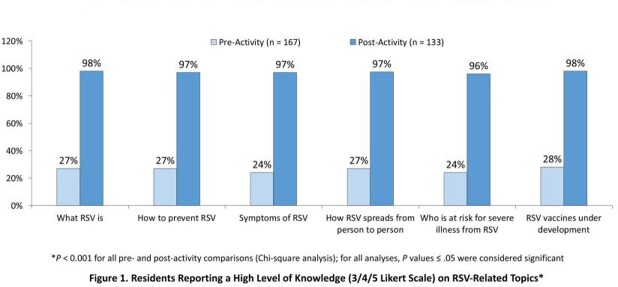

**Figure:**
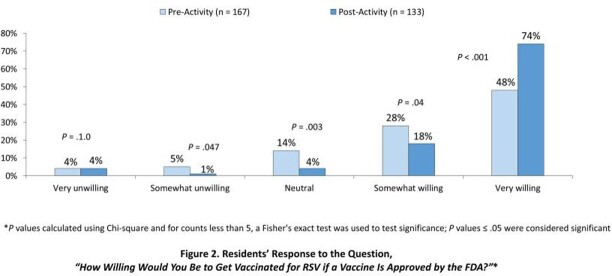

**Conclusion:**

While residents of LTCFs recognize the importance of RSV testing and prevention, they exhibit low levels of knowledge about RSV-related topics, lack of confidence in reducing the spread of disease, discomfort in raising issues regarding vaccination with their HCP, and unwillingness to get the RSV vaccine once available. Educational sessions can help to narrow knowledge gaps, increasing the potential for changes in behavior related to RSV prevention.

**Disclosures:**

**Stefan Gravenstein, MD, MPH**, Genentech: Advisor/Consultant|Genentech: Grant/Research Support|Janssen: Advisor/Consultant|Janssen: Honoraria|Longeveron: Honoraria|Merck: Honoraria|Moderna: Advisor/Consultant|Pfizer: Advisor/Consultant|Pfizer: Grant/Research Support|Sanofi: Grant/Research Support|Sanofi: Honoraria|Seqirus: Grant/Research Support|Seqirus: Honoraria.

